# Polygyny with mate fidelity in the saxicolous frog

**DOI:** 10.1038/s42003-020-01255-y

**Published:** 2020-09-22

**Authors:** Caitlin Karniski

**Affiliations:** Communications Biology, https://nature.com/commsbio

## Abstract

While polygyny is common among vertebrates, polygyny with mate fidelity has not yet been demonstrated in amphibians. A recent study by Fábio de Sá and colleagues shows that single male saxicolous frogs share a breeding territory with two females and mate multiple times with them over the course of a breeding season. These authors attribute the evolution of this mating system to the intense competition for territories and mates when access to these resources is scarce.

Vertebrates demonstrate a wide range of mating systems—from promiscuous mating, where both sexes mate with multiple partners, to monogamy. Polygyny, in which a single male mates with multiple females, also exhibits variation in the level of reproductive fidelity within or across breeding seasons. While polygyny is common across vertebrates, and among tetrapods specifically, this mating system has not yet been conclusively shown to occur with mate fidelity in amphibians.

A recent study^1^ led by Fábio de Sá of the University of Campinas reveals just that in the saxicolous frog *Thoropa taophora*. This work combines the use of behavioral observation in the rainforests of São Paulo state, Brazil and molecular parentage analyses to show that not only are the egg clutches of multiple females fertilized by a single male, but that these males also share their territories with these females over the course of a mating season.

While males aggressively defend their territories against cannibalizing conspecifics, the researchers found that males share their territories with two genetically unrelated females (and sometimes a third periphery female) and their egg clutches, and mate multiple times with both over the course of the 10-month breeding season. Further, the authors show that females within a breeding group demonstrate a dominance hierarchy, each with distinct behavioral tactics for mating, and that dominant females experience higher reproductive success.

The authors suggest that this mating system evolved due to a lack of available breeding sites, which leads to intense competition, both among males for territories and among females for access to males that defend these sites. At times when territory is scarce, as can be the case for the breeding sites located at freshwater seeps in São Paulo for the saxicolous frog, it may be more advantageous for a female to “share” a male at a superior breeding site rather than to be the sole female at a poor quality breeding territory.
